# Molecular detection and phylogenetic analyses of *Anaplasma* spp. in* Haemaphysalis longicornis* from goats in four provinces of China

**DOI:** 10.1038/s41598-021-93629-3

**Published:** 2021-07-08

**Authors:** Yaqun Yan, Kunlun Wang, Yanyan Cui, Yongchun Zhou, Shanshan Zhao, Yajun Zhang, Fuchun Jian, Rongjun Wang, Longxian Zhang, Changshen Ning

**Affiliations:** 1 College of Veterinary Medicine, Longzihu Campus of Henan Agricultural University, No. 15 Longzihu University Area, Zhengdong New District, Zhengzhou, 450046 People’s Republic of China; 2 School of Biotechnology and Food, Shangqiu Normal University, Shangqiu, 476000 People’s Republic of China

**Keywords:** Parasitology, Pathogens

## Abstract

*Anaplasma* species, which are distributed worldwide, are gram-negative obligate intracellular tick-borne bacteria that pose a threat to human and animal health. *Haemaphysalis longicornis* ticks play a vital role as vectors in the transmission of *Anaplasma* pathogens. However, the *Anaplasma* species carried by *H. longicornis* in China are yet to be characterized. In this study, 1074 *H. longicornis* specimens were collected from goats in four provinces of China from 2018 to 2019 and divided into 371 sample pools. All tick sample pools were examined for the presence of *Anaplasma* species via nested PCR amplification of 16S ribosomal RNA, major surface protein 4 (*msp4*), or citric acid synthase (*gltA*) genes, which were sequenced to determine the molecular and phylogenetic characteristics of the isolates. The overall *Anaplasma* spp-positive rate of *H. longicornis* was determined to be 26.68% (99/371). The percentage prevalence of *A. phagocytophilum*-like1, *A. bovis*, *A. ovis*, *A. marginale*, and *A. capra* were 1.08% (4/371), 13.21% (49/371), 13.21% (49/371), 1.35% (5/371), and 10.24% (38/371), respectively, and the co-infection rate of two or more types of *Anaplasma* was 6.47% (24/371). Phylogenetic analyses led to the classification of *A. phagocytophilum* into an *A. phagocytophilum*-like1 (*Anaplasma* sp. Japan) group. *Anaplasma bovis* sequences obtained in this study were 99.8–100% identical to those of an earlier strain isolated from a Chinese tick (GenBank accession no. KP314251). *Anaplasma ovis* sequences showed 99.3–99.6% identity to an *A. ovis* human strain identified from a Cypriot patient (GenBank accession no. FJ460443). Only one *msp4* sequence of *A. marginale* was detected and was grouped with those of other *A. marginale* isolates, and these *A. capra* isolates obtained in this present study may be zoonotic. The detection and characterization of four *Anaplasma* species in *H. longicornis* in this study have added to the current knowledge of the parasite and provided data on multiple *Anaplasma* species with veterinary and medical significance from four provinces of China.

## Introduction

The genus *Anaplasma* includes obligate intracellular parasitic pathogens transmitted by ticks, some of which are zoonotic and cause anaplasmosis in humans and animals^1^. At present, the genus mainly comprises *A. phagocytophilum*, *A. ovis*, *A. bovis*, *A. marginale*, *A. platys*, *A. centrale*, and the recently discovered *A. capra*^2,3^. Among the *Anaplasma* species, *A. phagocytophilum* not only infects neutrophil granulocytes of rodents and ruminants, such as sheep, goats, cattle and deer, but also humans^4,5^. *Anaplasma phagocytophilum* reportedly causes human granulocytic anaplasmosis (HGA) with symptoms of fever, headache, discomfort, myalgia, leukopenia, and thrombocytopenia^6^ and have been identified in North America, Europe, and Asia^7^. More recently, strains genetically related to *A. phagocytophilum*, i.e., *A. phagocytophilum*-like1: *Anaplasma* sp. and *A. phagocytophilum*-like2: *Anaplasma* sp., were identified in Japan and China, respectively^8–10^. *Anaplasma bovis* usually parasitizes monocytes and causes disease in small mammals and ruminants with symptoms of fever, weight loss, and ultimately, the possible death of cattle^11^. This pathogenic bacterium has mainly been identified in African and Asian countries, including Tunisia, China, and Japan^1^. *Anaplasma ovis*, a pathogen that parasitizes erythrocytes, is globally distributed and considered the most common cause of anaplasmosis in small ruminants, inducing fever, fatigue, anorexia, reduced milk production, and abortion, although related mortality rates are relatively low^12^. In addition, *A. ovis* is a potential human pathogen, and so far the only known human anaplasmosis case associated with this species was identified in 2007 in a Cypriot patient, who presented with fever, lymphadenopathy, and hepatosplenomegaly^6^. *Anaplasma marginale* are erythrocytic parasites causing bovine anaplasmosis that are transmitted by *Ixodes* sp., *Dermacentor* sp., *Rhipicephalus* sp., and *Haemaphysalis* sp., ticks, and clinical signs may include fever, weight loss, abortion, lethargy, and icterus^13–15^. Recently discovered in China, *Anaplasma capra* is a novel tick-transmitted pathogen, and its vectors and target cell types remain to be elucidated^3^. The pathogen infects several ruminants, such as goats, sheep, and deer (*Hydropotes inermis argyropus*), as well as humans^16^.

*Haemaphysalis longicornis* is distributed throughout China, with reports of infection in a variety of host animals, including dogs, goats, cattle, and sheep^17^. Some pathogens have been detected in *H. longicornis*, for instance, *Anaplasma* spp., *Rickettsia conorii*, *Babesia ovata*, and *Ehrlichia canis*^18,19^. However, limited information is available about *Anaplasma* app in *H. longicornis*. The main goal of the study was to identify the *Anaplasma* species carried by *H. longicornis* in China with a view to generating further information and enriching the available data on these pathogens, which may provide the basis for prevention and control strategies.

## Materials and methods

### Tick collection and identification

**Figure 1:**
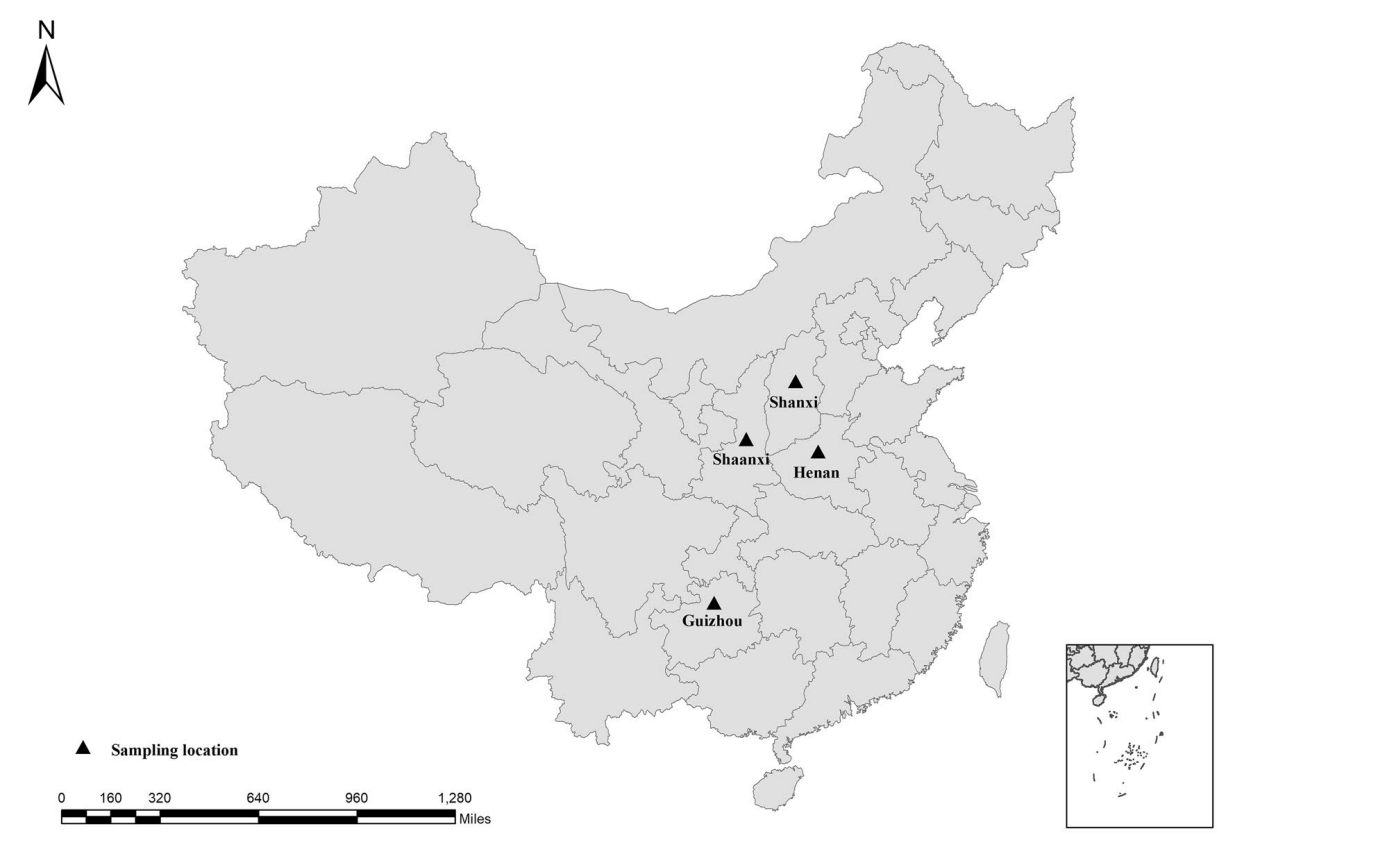
Geographic map of the sampling locations of China. The figure was originally designed by the authors under the software ArcGIS 10.2. The original vector diagram imported in ArcGIS was adapted from Natural Earth (http://www.naturalearthdata.com).

Our study was conducted at four localities in China (Fig. 1). Shaanxi (34° 40′ N; 107° 27′ E), Shanxi (34° 49′N, 111° 15′ E), and Henan (34° 20′ N; 111° 48′ E) have warm temperate climates with 500–800 mm average annual precipitation, while Guizhou (26° 44′ N; 106° 27′ E) has a subtropical humid and mild climate and average annual precipitation of 1129 mm. Between 2018 and 2019, 39, 3, 4 and 36 goats were randomly selected from Shaanxi, Shanxi, Henan and Guizhou, respectively. Tweezers were used to collect 10–15 adult ticks from the ears, face, and neck of each goat (once per goat) and were placed in a 10-mL centrifuge tube and stored in 70% ethanol until identification. Informed consent was obtained from the animal owners for the collection of tick sample. Family, genus, developmental stage, and species of all collected ticks were identified based on morphology^20,21^. Tick identity was further confirmed by analyzing 400 bp of the 16S rRNA gene using 16S − 1 and 16S + 1 primers^22^. All the ticks collected from goats were grouped by species, sex, and sampling site into pools^23^ and the 1074 *H. longicornis* specimens were divided into 371 sample pools. All sample pools were individually placed into tubes containing 70% ethanol and stored at − 4 °C prior to DNA extraction.

### DNA extraction

First, ticks were washed sequentially with 30%, 50%, and 70% absolute ethanol and distilled water for 5 min each time and dried on sterile filter paper. Next, they were ground in a grinder with liquid nitrogen. DNA was extracted according to the manufacturer’s instructions for the Universal Genomic DNA Kit (CwBio, Beijing, China) and eluted in a final volume of 100 μL. Extracted DNA was stored at − 20 °C until experimental use.

### PCR amplification

To assess whether the *H. longicornis* specimens were infected with *Anaplasma* species, nested PCR assays targeting the 16S rRNA gene of *A. bovis*, *A. phagocytophilum* and related strains, the major surface protein 4 (*msp4*) gene of *A. ovis* and *A. marginale*, and the citric acid synthase (*gltA*) gene of *A. capra* were performed for all *H. longicornis* sample pools. The first PCR reaction system contained 4 µL dNTP mixture (2.5 mmol L^−1^), 2.5 μL 10 × LA PCR Buffer (Mg^2+^ Plus), 0.5 μL forward and reverse primers (20 pmol each), 1.25 U LA *Taq* DNA Polymerase (5 U μL^−1^) (TaKaRa, Dalian, China), 1 μL raw DNA template, and 16.25 µL distilled water. The final amplification reaction was conducted in a 25 μL volume containing 2.0 μL PCR products, 0.75 U *Taq* DNA polymerase (5 U μL^−1^) (TaKaRa, Dalian, China), 2.5 μL 10 × PCR buffer (Mg^2+^ Plus), 2.0 μL dNTPs (concentration of 2.5 mM), 0.5 μL primers (20 pmol each), and 17.35 μL distilled water. All PCR reactions were conducted using an ABI 2720 thermal cycler instrument (Life Technologies Holdings Pte Ltd., Singapore). The primers and amplification conditions used are listed in Table 1. In each PCR assay, DNA samples that had been sequenced and kept in the laboratory from sheep positive for *A. phagocytophilum*, *A. bovis*, *A. ovis*, *A. capra* and cattle positive for *A. marginale*, were run as the positive controls and distilled water as the negative control. PCR products (5 μL) were separated via electrophoresis on a 1.0% agarose gel and visualized via UV transillumination following ethidium bromide staining.

**Table 1:** Primers and amplification conditions for PCR detection of *Anaplasma* spp. in *H. longicornis*.

Pathogens	Target gene	Primer (5′–3′)	Amplicon (bp)	Thermocycler program				Cycles	Final extension	References
				Denaturation Annealing						
*A. phagocytophilum *and related strains	16S rRNA	EE1: TCCTGGCTCAGAACGAACGCTGGCGGC	1430	94 °C 5 min	94 °C 30 s	55 °C 30 s	72 °C 30 s	35	72 °C 10 min	^24^
		EE2: GTCACTGACCCAACCTTAAATGGCTG								
		SSAP2f: GCTGAATGTGGGGATAATTTAT	641	94 °C 5 min	94 °C 35 s	55 °C 40 s	72 °C 40 s	40	72 °C 10 min	^25^
		SSAP2r: ATGGCTGCTTCCTTTCGGTTA								
*A. bovis*	16S rRNA	EE1: TCCTGGCTCAGAACGAACGCTGGCGGC	1430	94 °C 5 min	94 °C 30 s	55 °C 30 s	72 °C 30 s	35	72 °C 10 min	^24^
		EE2: GTCACTGACCCAACCTTAAATGGCTG								
		AB1f: CTCGTAGCTTGCTATGAGAAC	551	94 °C 5 min	94 °C 30 s	55 °C 30 s	72 °C 30 s	40	72 °C 10 min	^25^
		AB1r: TCTCCCGGACTCCAGTCTG								
*A. ovis*	*msp4*	AMOf: GCTCCCTACTTGTTAGTGG AMOr: TTAGCTGAACAGGAATCTTG	795	94 °C 5 min	94 °C 30 s	58 °C 30 s	72 °C 1 min	25	72 °C 7 min	^26^
		MSP4f: CAAGCAGAGAGACCTCGTAT	584	94 °C 5 min	94 °C 30 s	57 °C 30 s	72 °C 1 min	36	72 °C 7 min	
		MSP4r: GGCTTTTGCTTCTCCGGG								
*A. marginale*	*msp4*	MSP45: GGGAGCTCCTATGAATTACAGAGAATTGTTTAC	852	95 °C 5 min	95 °C 30 s	57 °C 30 s	72 °C 45 s	36	72 °C 7 min	^27^
		MSP43: CCGGATCCTTAGCTGAACAGGAATCTTGC								
*A. capra*	*gltA*	acagltaf1:GCGATTTTAGAGTGYGGAGATTG	1031	94 °C 5 min	94 °C 45	55 °C 45 s	72 °C 1 min	25	72 °C 7 min	^28^
		acagltar1:TACAATACCGGAGTAAAAGTCAA								
		acagltaf2:TCATCTCCTGTTGCACGGTGCCC	594	94 °C 5 min	94 °C 45 s	60 °C 45 s	72 °C 1 min	30	72 °C 7 min	
		acagltar2:CTCTGAATGAACATGCCCACCCT								

### Sequence and phylogenetic analyses

PCR products were sequenced by a commercial company (TSINGKE, Beijing, China). Sequence accuracy was verified via bidirectional sequencing, and sequences were identified and analyzed using BLASTN (http://www.ncbi.nlm.nih.gov/BLAST) and CLUSTALW 2.0.10 (https://www.ebi.ac.uk/Tools/msa/clustalo/) programs. To ascertain the phylogenetic placement of *Anaplasma* spp. identified in this study, a phylogenetic tree was constructed based on the sequence distance method using the Maximum likelihood method with the best evolutionary model of MEGA 7.0 (http://www.megasoftware.net). Confidence values for each branch of the resulting tree were confirmed by bootstrap analysis with 1000 replicates.

### Statistical analysis

The infection rates of *Anaplasma* in sample pools of *H. longicornis* from different sites and different sexes were compared using the chi-square test in SPSS version 22.0 (SPSS, Inc., Chicago, IL, USA). Data were considered significant at *P* < 0.05.

### Nucleotide sequence accession numbers

The GenBank accession numbers obtained in this study were as follows: MN097866 and MN097867 for *A. phagocytophilum*-like1, MK991952 to MK991955 for *A. bovis*, MK991961 to MK991963 for *A. ovis*, MW772454 for *A. marginale*, MK991956 to MK991960 for *A. capra*, and the accession numbers MN956525 to MN956527 for *H. longicornis*.

### Ethics statement

This study was carried out in accordance with the Chinese Laboratory Animal Administration Act (1988) after it was reviewed and its protocol was approved by the Research Ethics Committee of Henan Agricultural University. Appropriate permission was gained from the animal owners before the collection of ticks.

## Results

### Rates of positivity for *Anaplasma* species

All ticks taken from goats were identified as *H. longicornis*. As shown in Table 2, *A. phagocytophilum*-like1, *A. bovis*, *A. bovis*, *A. marginale*, and *A. capra* were detected in *H. longicornis* collected from Shaanxi, Shanxi, Guizhou, and Henan provinces. From the 371 sample pools tested, 99 (26.68%) were positive for *Anaplasma* species. The infection percentages of *A. phagocytophilum*-like1, *A. bovis*, *A. ovis*, *A. marginale*, and *A. capra* were 1.08% (4/371), 13.21% (49/371), 13.21% (49/371), 1.35% (5/371), and 10.24% (38/371), respectively. *Anaplasma* infection percentages in *H. longicornis* collected at different sampling sites varied from 0% (0/9) to 43.59% (68/156) and were significantly different between sites (*P* < 0.05).

**Table 2:** Rates of positivity for *Anaplasma* species in ticks grouped by sex and sampling location.

Group	Anaplasma infection rate (%)					
	*A. phagocytophilum*-like1		*A. bovis*	*A. ovis*	*A. capra*	*A. marginale*
Sampling location	Shaanxi	0.57 (1/175)	2.29 (4/175)	10.29 (18/175)	6.86 (12/175)	0 (0/175)
	Guizhou	0.64 (1/156)	28.85 (45/156)	12.18 (19/156)	14.10 (22/156)	0 (0/156)
	Shanxi	11.1 (1/9)	0 (0/9)	0 (0/9)	0 (0/9)	0 (0/9)
	Henan	3.2 (1/31)	0 (0/31)	38.7 (12/31)	12.9 (4/31)	16.13 (5/31)
Sex	Female	1.66 (4/241)	9.54 (23/241)	11.20 (27/241)	8.30 (20/241)	2.07 (5/241)
	Male	0 (0/130)	20.00 (26/130)	16.92 (22/130)	13.85 (18/130)	0 (0/130)
Total		1.08 (4/371)	13.21 (49/371)	13.21 (49/371)	10.24 (38/371)	1.35 (5/371)

We additionally observed a significant difference in *Anaplasma* infection between the sexes (0.01 < *P* < 0.05), specifically, 25.31% (61/241) and 36.92% (48/130) in female and male ticks, respectively. Moreover, the 13.21% infection rates determined for *A. bovis* and *A. ovis* were significantly higher than those for *A. phagocytophilum*-like1 (1.08%) (0.01 < *P* < 0.05) and *A. marginale* (1.35%) (0.01 < *P* < 0.05) but only slightly higher than that of *A. capra* (10.24%) (*P* > 0.05). As shown in Table 3, 24 samples were infected with two or more types of *Anaplasma* concurrently, and the co-infection rate of *Anaplasma* was 6.47% (24/371).

**Table 3:** Co-infection rate of *Anaplasma* in *H. longicornis.*

Pathogen	*A. bovis* + *A. capra*	*A. ovis* + *A. capra*	*A. bovis* + *A. ovis*	*A. ovis* + *A. marginale*	*A. phagocytophilum-like1* + *A. bovis* + *A. capra*	*A. phagocytophilum-like1* + *A. ovis* + *A. capra*
Mixed infection rate (%)	2.7 (10/371)	0.3 (1/371)	2.7 (10/371)	0.3 (1/371)	0.3 (1/371)	0.3 (1/371)

### Phylogenetic analysis

Phylogenetic analysis was performed by aligning sequences obtained in this study with sequences available in GenBank from selected ticks and *Anaplasma* spp. isolated from ticks, ruminants and humans. The three *H. longicornis* 16S rRNA sequences displayed 98.5–99.8% identity to each other and showed 98.8–100% identity to *H. longicornis* collected in China (MH024508).

**Figure 2:**
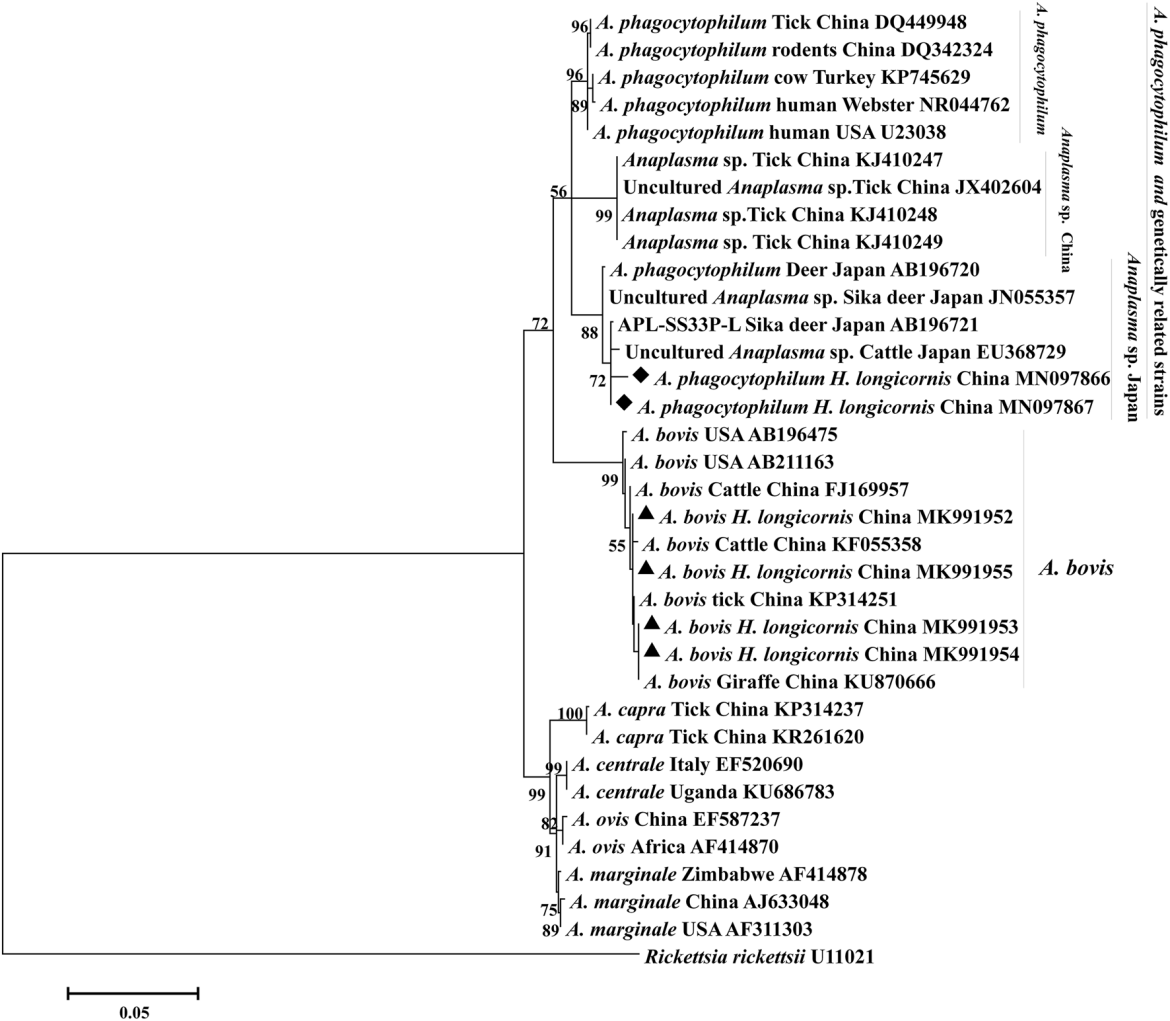
Phylogenetic relationship between 16S rRNA gene sequences of *Anaplasma* spp. Phylogenetic tree constructed based on partial 16S rRNA gene sequences of *A. phagocytophilum* and *A. bovis* using the Maximum likelihood method with the best evolutionary model and bootstrap analysis of 1000 replicates. The *A. phagocytophilum* like strain and *A. bovis* sequences obtained in this study are indicated by black diamonds and Black triangles, respectively. Values lower than 50% are hidden.

The genotypes (MN097866, MN097867) obtained in this study were classified as strains genetically related to the *A. phagocytophilum* (*Anaplasma* sp. Japan) cluster (Fig. 2). Sequencing the 16S rRNA gene showed that the two strains from *H. longicornis,* which were genetically related to *A. phagocytophilum*, displayed 98.7% identity to each other*.* The 16S rRNA gene sequences from the four *A. bovis* found in *H. longicornis* displayed 99.6–100% identity to each other and 99.8–100% identity to a tick strain (KP314251) found in China. Furthermore, they belonged to the same clade as isolates from Chinese cattle, giraffe (*Giraffa camelopardalis giraffa*), and tick (KF055358, KU870666, KP314251, respectively) (Fig. 2).

**Figure 3:**
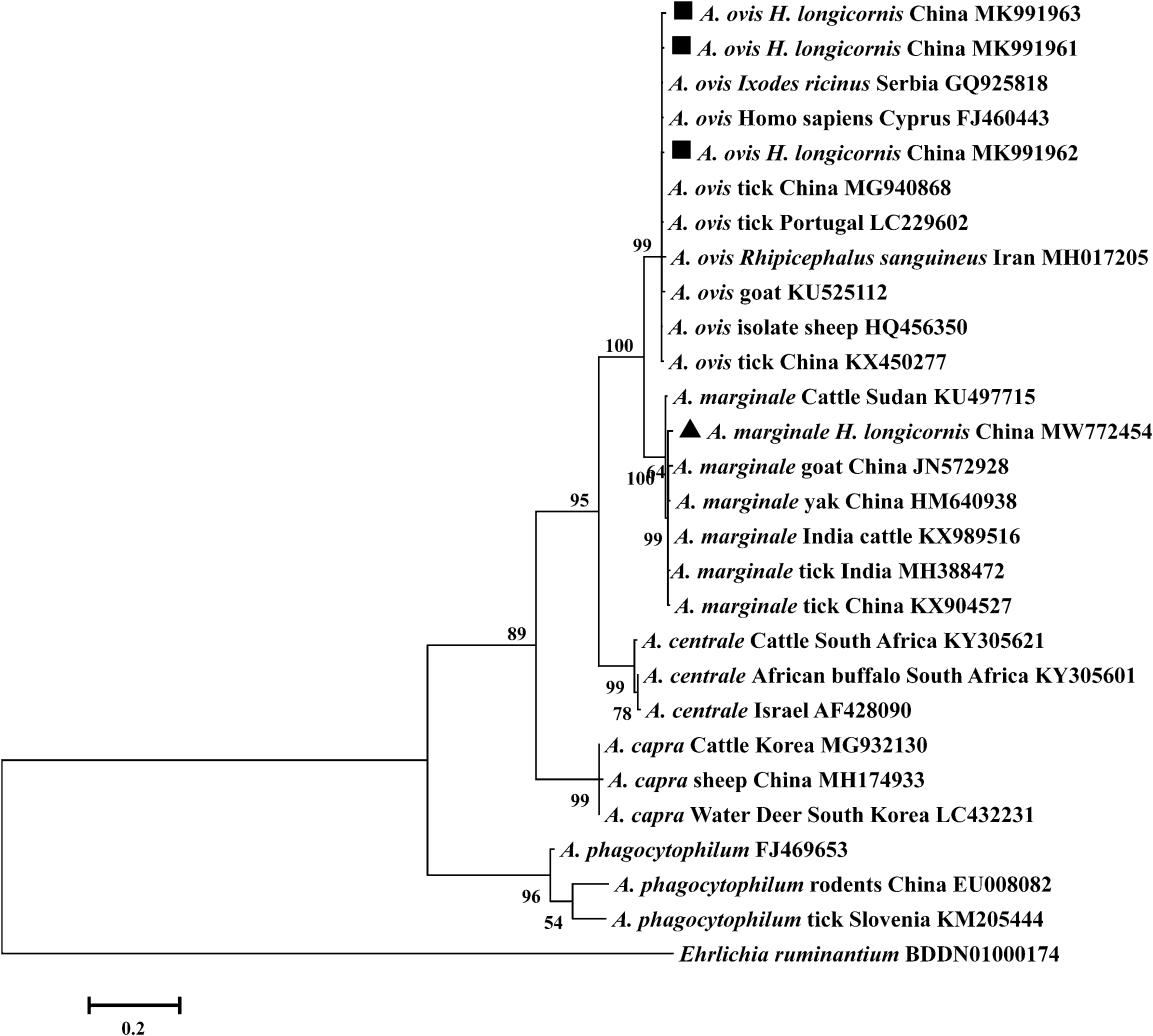
Phylogenetic relationships between gene sequences of *Anaplasma* spp. Phylogenetic tree constructed based on partial *msp4* gene sequences of *A. ovis* and *A. marginale* using the Maximum likelihood method with the best evolutionary model and bootstrap analysis of 1000 replicates. The *A. ovis* and *A. marginale* sequences obtained in this study are indicated by black squares and Black triangle, respectively. Values lower than 50% are hidden.

**Figure 4:**
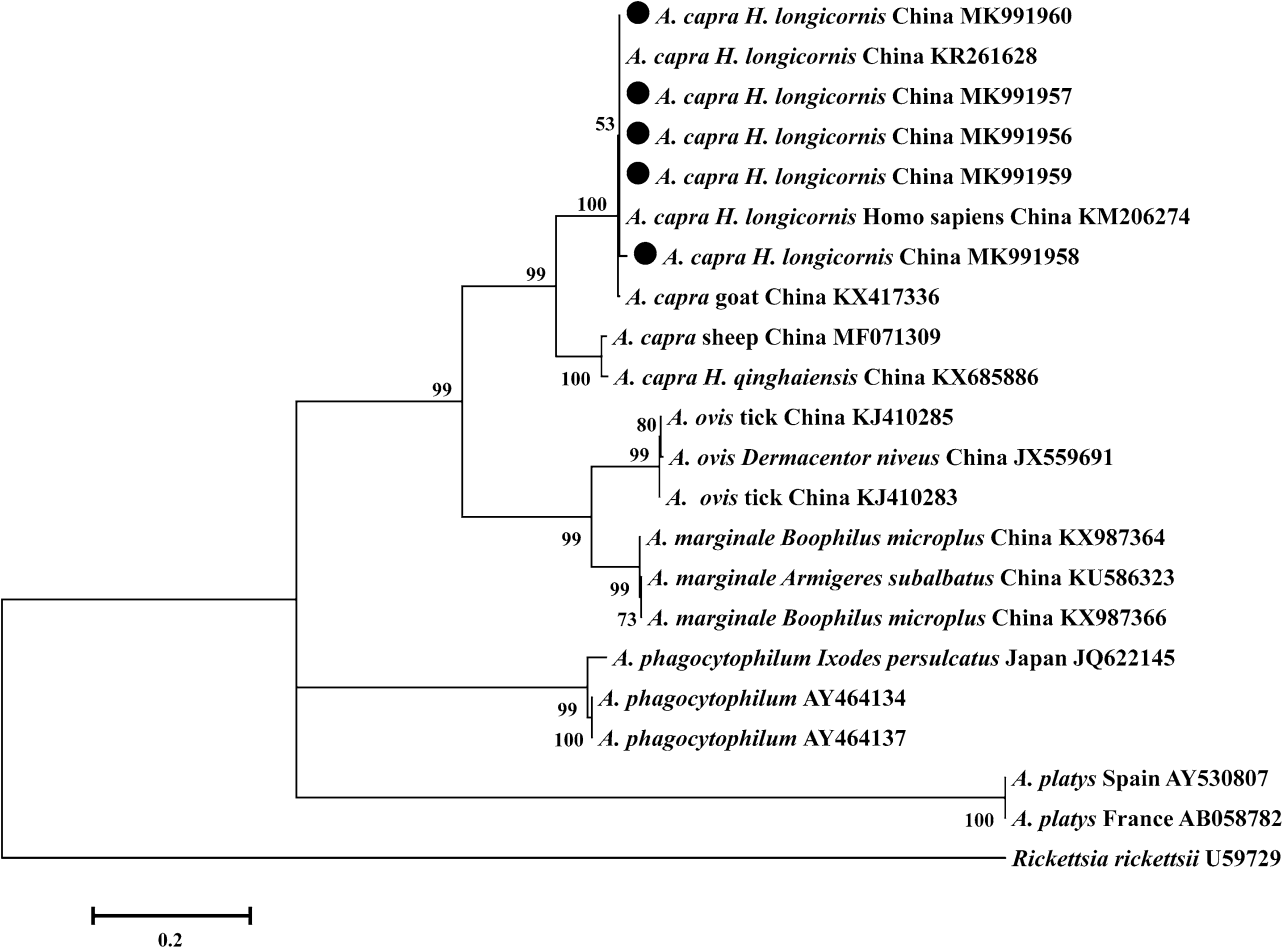
Phylogenetic relationships between *gltA* gene sequences of *Anaplasma* spp. Phylogenetic tree based on partial *gltA ge*ne sequences of *A. capra* by using the Maximum likelihood method with the best evolutionary model and bootstrap analysis of 1000 replicates. Black circles indicate the sequences obtained in this study.

The three *A. ovis msp4* sequences were 98.9–99.3% identical to each other and 99.3–99.6% identical to a human *A. ovis* strain from Cyprus (FJ460443) grouped within the same clade (Fig. 3). The only *msp4* sequence of *A. marginale* identified had a homology of 98.9–99.9% with other *A. marginale* isolates and was therefore placed in the same group (Fig. 3). The five *A. capra gltA* sequences identified in *H. longicornis* were 98.4–100% identical to a human isolate from China (KM206274). Phylogenetic analysis showed the genotypes obtained in this study were classified in *A. capra* cluster. (Fig. 4).

## Discussion

Previous studies have demonstrated that different kinds of ticks, such as *Haemaphysalis qinghaiensis*, *Dermacentor* silvarum, *D. nuttalli* and so on, existed different degrees of infection of *Anaplasma* pathogen^29,30^. However, a limited number of investigations into these pathogens and their mechanisms of infection have been conducted in China. In this study, we identified and characterized five *Anaplasma* species that may present a potential threat to human and animal health, *A. phagocytophilum*, *A. bovis*, *A. ovis*, *A. marginale*, and *A. capra*, isolated from *H. longicornis* sampled in four provinces of China.

In recent years, two variants genetically related to *A. phagocytophilum* have been independently detected in surveys undertaken in Asia. For instance, *A. phagocytophilum-*like 1 (*Anaplasma* sp.-Japan) has been discovered in sika deer (Cervus nippon yesoensis), cattle, and ticks infesting ruminants in Japan (*Ixodes persulcatus*, *I. ovatus*, *Hyalomma megaspinosa*)^16,31^. while *A. phagocytophilum*-like 2 (*Anaplasma* sp.-China) has been recently detected in cattle, goats, and *H. asiaticum* ticks infesting ruminants in China^9,32,33^. In this study, *A. phagocytophilum-*like1 (*Anaplasma* sp.-Japan) has been identified in *H. longicornis* (Fig. 2), and the *H. longicornis* infection percentages of this variant in Henan, Shaanxi, Shanxi, and Guizhou provinces were determined to be 3.2% (1/31), 0.58% (1/171), 11.1% (1/9), and 0.65% (1/155), respectively. This suggests that *H. longicornis* may be a common transmission medium for *A. phagocytophilum*-like strains in these four Chinese provinces. Therefore, in the future, further studies into the distribution of *A. phagocytophilum* and *A. phagocytophilum*-like strains in tick species and infested ruminants or other animals should be carried out in parts of China.

*Anaplasma bovis,* which was first reported in cattle, has been shown to infect circulating monocytes and tissue macrophages^34,35^. To date, *A. bovis* has been isolated from dogs, sheep, goats, and wild deer (*Cervus nippon nippon*) in addition to vector ticks, including *H. longicornis*, *Rhipicephalus appendiculatus*, and *H. qinghaiensis*, indicating they have a broad host range^30,36,37^. The *A. bovis* infection rate in *H. longicornis* was 13.39% in our study, and related studies have also been detected in Japan (12.0%), Korea (0.4%), Shenyang (0.6%), Heilongjiang province (0.7%)^25,38–40^. These findings signify that *H. longicornis* may be a vector for *A. bovis*, both in China and many other parts of the world. Moreover, the *A. bovis* infection incidence of *H. longicornis* ticks was significantly higher in Guizhou than the other sampling sites (*P* < 0.05), possibly due to the different geographical conditions. Guizhou, where *Anaplasma* bacteria are widespread, is the natural focal area of anaplasmosis^41^; moreover, it is a subtropical region with rich vegetation, which is suitable for a variety of host animals of *Anaplasma.* However, there was no *A. bovis* DNA in ticks from Shanxi and Henan in the present study. The putative absence of *A. bovis* in ticks at the two sampling localities is probably a reflection of the small numbers of ticks tested. The *A. bovis* isolates obtained in this study were 99.8–100% identical to other isolates from different hosts in other parts of China, and they were located on the same clade (Fig. 2). *Anaplasma bovis* has experienced genetic stability and the absence of geographical and host isolation in China based on 16S rRNA gene, which is consistent with the earlier report of Yang et al.^42^. Therefore, more experiments should be performed to explore the geographical and host isolation of *A. bovis*.

*Anaplasma ovis* is widely distributed in North America, Asia, Africa, and Europe and has been identified in sheep, goats, *Dermacentor abaensis*, and *Haemaphysalis tibetensis*^43,44^. However, there have been no reports of *A. ovis* being carried by *H. longicornis* documented to date. In Shaanxi, Shanxi, Guizhou, and Henan, the overall *A. ovis* infection percentage of *H. longicornis* was determined to be 13.9%, which is higher than that of *H. qinghaiensis* in Qinghai (4.0%)^30^. In addition, *A. ovis* was detected at all sampling sites except Shanxi, which may be attributable to the different geographical environment or limited number of samples. In the current study, four *msp4* gene sequences of *A. ovis* were identified in *H. longicornis* ticks. And these four strains also showed high similarities to those isolates previously obtained from China, Portugal, Serbia, Cyprus and Iran, indicating low diversity of *A. ovis* in the study ticks. Furthermore, *A. ovis* isolates of *H. longicornis* in this investigation were closely related to a human isolate from Cyprus (FJ460443), suggesting this pathogen may post a threat to public health (Fig. 3).

It has been documented that ticks of *Rhipicephalus* sp. and *Haemaphysalis* sp. are responsible for the transmission of *A. marginale* to a variety of vertebrate hosts^45^. The prevalence of *A. marginale* in *H. longicornis* in the present study was 1.35% (5/371), and the detection result is consistent with previous reports to a certain extent. Even though *A. marginale* populations worldwide typically infect cattle, causing bovine anaplasmosis, and have a significant economic impact on the cattle industry^46^, the species has also been detected in sheep^47^ and goats^48,49^. Because the ticks in this study were collected from goats, it is necessary to explore the presence of *A. marginale* in goats and other animals at the sampling sites.

*Anaplasma capra* is an emerging zoonotic pathogen that has been detected in sheep, goats, *H. qinghaiensis*, *I. persulcatus*, and humans^16,30^. In this study, 10.38% of *H. longicornis* individuals were positive for *A. capra*, which is a higher percentage than found for *A. capra* infection of *H. longicornis* ticks in Qinghai (0.03%), Korea (0.03%), and Shandong (0.84%)^30,39,50^. These discrepancies may be attributed to the variations in climate, vegetation, hosts, tick activity, time of sampling, and detection methods. The *A. capra gltA* gene isolated in our experiments exhibited high genetic diversity relative to *A. capra* isolated (KR261626, KX417324, and KM206274) in earlier surveys. When we phylogenetically analyzed the *A. capra* isolates based on the *gltA* gene, the all sequences of *A. capra* fell into two clades. Additionally, *A. capra* isolates obtained in this study and other *A. capra* sequences isolated from ticks and ruminants available in the GenBank fell into the same clade with isolate from human. Therefore, these *A. capra* isolates obtained in this present study may be zoonotic (Fig. 4).

Our study had a number of limitations that should be acknowledged. While the results highlight the current status of *Anaplasma* infections in *H. longicornis* in four provinces of China, investigations in other parts of China have yet to be conducted. Moreover, ticks were not collected from the environment and other hosts, and tick larvae and nymphs were not used in the experiments. Although the present study has revealed the current status of *H. longicornis* tick infestation with *Anaplasma* spp. in the investigated areas, the specific biological vector for the individual *Anaplasma* species need to be further studied by transmission experiments. In addition, the infections of *Anaplasma* species in different hosts (including animals and humans) and regions should be investigated to understand the true impact of anaplasmosis.

## Conclusion

Our investigation revealed a prevalence of *A. phagocytophilum-*like 1 (*Anaplasma* sp.-Japan), *A. bovis*, *A. ovis*, *A. marginale* and *A. capra* in *H. longicornis* from four provinces in China. These findings add to the existing body of knowledge on the public health risk of *H. longicornis* carrying *Anaplasma* pathogens and may provide a basis for strategies to prevent and control anaplasmosis.
